# Supplementary material for: Empirical methods for controlling false positives and estimating confidence in ChIP-Seq peaks
Source: BMC Bioinformatics. 2008 Dec 5;9:523. doi: 10.1186/1471-2105-9-523 (PMC2628906; doi:10.1186/1471-2105-9-523)
Supplement: Additional File 1 — A variety of html documents from the USeq web site detailing the available applications, their best usage, output file type descriptions, command line menus, etc. [file 1471-2105-9-523-S1.zip › USeqUserGuides/JavaDoc.zip!/JavaDoc/meme/MemeR.html]

MemeR


|  |  |  |
| --- | --- | --- |
| **Overview** \| **Package** \| **Class** \| **Use** \| **Tree** \| **Deprecated** \| **Index** \| **Help** | |  |
| **PREV CLASS**   **NEXT CLASS** | **FRAMES**    **NO FRAMES**     **All Classes** |  |
| SUMMARY: NESTED \| FIELD \| CONSTR \| METHOD | DETAIL: FIELD \| CONSTR \| METHOD |  |


---


## meme Class MemeR

```
java.lang.Object
  meme.MemeR
```

---

``` public class MemeR extends java.lang.Object ```

Main, wRapper for meme geared toward processing multiple rounds of selex data.
This is a major revision of MemeR, I've equipped it for mass data processing. Multiple files can be submitted. Each
is searched for the presence of motifs using UCSD's meme program. These motifs are stored and then used to search the
the sequences in each file for the presence of the motif, hits above a user defined cut off. Multiple results and
summary tables are printed and saved. The program works by first firing the main method in MemeR which in turn creates
a MemeResults object that will hold most of the data generated by the program. User inputs are processed asking for:
1)parsed selex multi-FASTA files separated by a comma 2)meme command line arguments 3)a meme cutoff score 4)a
directory to save the result files 5) a basename to use in naming the result files. UCSD's meme is then fired for each
file entered. A MemeParser is created to parse the meme output, a MemeMotif is created for each motif parsed from the
meme output. Once the motifs have been created. A MotifScanner object is created from a motif and a fasta file and
executes a search for motif hits. A MotifSearchResult object is used to hold the results from this search. If there
were any hits, these are stored as an array of MotifHit objects in the MotifSearchResult object. So here's the flow
MemeR->MemeParser->MemeMotif(s) MemeMotif->MotifScanner->MotifSearchResult->MotifHit(s)
Lastly, several MemeResults methods are called from MemeR to print various reports/ summaries to the screen and also
write them to file. These include a summary of the motifs identified, a summary table of motif hits in each file,
and a crude printed graph showing the percentage of sequences in each multi-fasta file that contained each motif.
nix@uclink.berkeley.edu 22 Oct 2004 MemeR(0.2.1)

---

**Constructor Summary**

`MemeR()`


| **Method Summary** | |
| --- | --- |
| `void` | `fireMeme()` |
| `void` | `getUserInputs()`             Uses JOptionPane dialog input boxes to get user inputs |
| `static void` | `main(java.lang.String[] args)` |
| `static java.lang.String` | `makeInputDialog(java.lang.String title, java.lang.String comment, java.lang.String suggestion)` |
| `void` | `searchDataFiles()` |

**Methods inherited from class java.lang.Object**

`equals, getClass, hashCode, notify, notifyAll, toString, wait, wait, wait`

**Constructor Detail**

### MemeR

```
public MemeR()
```


**Method Detail**

### main

```
public static void main(java.lang.String[] args)
```

---


### fireMeme

```
public void fireMeme()
```

---


### searchDataFiles

```
public void searchDataFiles()
```

---


### getUserInputs

```
public void getUserInputs()
```

:   Uses JOptionPane dialog input boxes to get user inputs

---


### makeInputDialog

```
public static java.lang.String makeInputDialog(java.lang.String title,
                                               java.lang.String comment,
                                               java.lang.String suggestion)
```


---


|  |  |  |
| --- | --- | --- |
| **Overview** \| **Package** \| **Class** \| **Use** \| **Tree** \| **Deprecated** \| **Index** \| **Help** | |  |
| **PREV CLASS**   **NEXT CLASS** | **FRAMES**    **NO FRAMES**     **All Classes** |  |
| SUMMARY: NESTED \| FIELD \| CONSTR \| METHOD | DETAIL: FIELD \| CONSTR \| METHOD |  |


---
